# Nicotinamide Prevents the Plasticity Impairments and the Cognitive Dysfunction Caused by Bone Fracture in Older Mice

**DOI:** 10.1093/gerona/glae303

**Published:** 2025-01-17

**Authors:** Tatiana Guncay, Jorge Concha, Pedro Lobos, Jamileth More, Barbara Bruna, Daniel Sansores, Pamela Contreras, Daniela P Ponce, Julian Brañez, Gabriel Quiroz, Genaro Barrientos, Cecilia Hidalgo, Felipe Salech

**Affiliations:** Centro de Investigación Clínica Avanzada (CICA), Hospital Clínico Universidad de Chile, Santiago, Chile; Centro de Investigación Clínica Avanzada (CICA), Hospital Clínico Universidad de Chile, Santiago, Chile; Centro de Investigación Clínica Avanzada (CICA), Hospital Clínico Universidad de Chile, Santiago, Chile; Clinical and Molecular Pharmacology Program, Institute of Biomedical Sciences (ICBM), Faculty of Medicine, University of Chile, Santiago, Chile; Centro de Investigación Clínica Avanzada (CICA), Hospital Clínico Universidad de Chile, Santiago, Chile; Laboratory of Translational Psychiatry, Department of Neuroscience and Department de Psychiatry North, Universidad de Chile, Santiago, Chile; Centro de Investigación Clínica Avanzada (CICA), Hospital Clínico Universidad de Chile, Santiago, Chile; Centro de Investigación Clínica Avanzada (CICA), Hospital Clínico Universidad de Chile, Santiago, Chile; Centro de Investigación Clínica Avanzada (CICA), Hospital Clínico Universidad de Chile, Santiago, Chile; Centro de Investigación Clínica Avanzada (CICA), Hospital Clínico Universidad de Chile, Santiago, Chile; Servicio de Traumatología, Hospital San José, Santiago, Chile; Geroscience Center for Brain Health and Metabolism (GERO), Santiago, Chile; Physiology and Biophysics Program, Institute of Biomedical Sciences (ICBM), Faculty of Medicine, Universidad de Chile, Santiago, Chile; Physiology and Biophysics Program, Institute of Biomedical Sciences (ICBM), Faculty of Medicine, Universidad de Chile, Santiago, Chile; Center for Exercise, Metabolism and Cancer Studies, Faculty of Medicine, Universidad de Chile, Santiago, Chile; Centro de Investigación Clínica Avanzada (CICA), Hospital Clínico Universidad de Chile, Santiago, Chile; Geroscience Center for Brain Health and Metabolism (GERO), Santiago, Chile; (Biological Sciences Section)

**Keywords:** Aging, Animal model, Delirium, Memory, Synaptic plasticity

## Abstract

Postoperative delirium (POD), an acute cognitive dysfunction linked to morbidity and mortality, is characterized by memory impairments and disturbances in consciousness, particularly in patients aged 65 and older. Neuroinflammation and NAD+ imbalance are key mechanisms behind POD, leading to synaptic and cognitive deterioration. However, how surgery contributes to POD and neuroinflammation remains unclear, and effective treatments are lacking. Here we used a rodent model of bone fracture to examine the impact of surgery on synaptic plasticity, inflammation, and cognition. Additionally, we explored whether treatment with nicotinamide (NAM), a NAD+ precursor, reduced the neuroinflammation and metabolic imbalance caused by surgery. Female C57BL/6J mice aged 20–22 months underwent tibial fracture surgery and received pre- and post-surgery NAM treatment. Neuroinflammation, synaptic plasticity, and cognition were assessed 72 hours post-surgery via long-term potentiation (LTP) assays, dendritic spine counting, and behavioral tests (open field maze and Y-maze). Tibial fracture surgery decreased LTP, dendritic spine density, and hippocampal-dependent memory function, and increased hippocampal inflammatory markers (IL-1β mRNA, CD38, and SIRT1 protein content); NAM pretreatment prevented these changes. Given surgery’s adverse effects on LTP and dendritic spine density, we assessed cellular oxidative state and brain-derived neurotrophic factor (BDNF) protein levels. We found that surgery increased the oxidation of ryanodine receptor calcium channels (cellular redox sensors), and decreased BDNF protein levels; NAM supplementation mitigated both effects and prevented the cognitive decline and synaptic plasticity deficits while reducing inflammation post-surgery by lowering IL-1β and CD38 protein levels. We propose that the CD38 signaling pathway mediates these NAM protective effects.

Postoperative delirium (POD), characterized by acute disturbances in cognition and consciousness (1), is prevalent in up to 53% of surgical cases (2), leading to increased mortality (3), long-term cognitive decline (4), and higher healthcare costs (5). Despite its impact, there are no established pharmacological interventions to prevent delirium, due to limited understanding of its underlying mechanisms.

Of note, POD has a multifactorial origin, arising from a complex interplay of patient vulnerability (predisposing factors) and environmental stressors (precipitating factors) (6). Aging emerges as a prominent predisposing factor for POD; persons aged 65 years and older are particularly susceptible to POD due to significant chronic comorbidities and age-related frailty (7).

Fragility hip fractures, which account for many unplanned orthopedic surgeries in older adults (8), are frequently accompanied by POD (9). These fractures exemplify high-intensity injury in frail adults aged 65 and older; thus, 13%–70% of these patients experience POD (9). While POD-related cognitive disturbances are usually transient, up to half of older adults may suffer significant cognitive effects for up to 6 months post-discharge (4). This lasting impact likely results from a reduced physiological reserve to handle the stress of the injury and the subsequent surgery.

The pathophysiology of POD in geriatric patients with hip fractures remains unclear, with research focusing on neuroinflammation, neurotransmitters, and metabolic abnormalities (1). Current evidence suggests that abnormal neuroinflammatory responses and metabolic failures in the brain significantly contribute to delirium (10,11). Adults aged 65 and older with hip fractures are particularly susceptible to complications from the inflammatory response triggered by both the fracture and the surgery.

Recent studies have linked delirium with increased levels of pro-inflammatory cytokines, such as IL-1β, IL-6, and TNF-α, in serum (12,13) and cerebrospinal fluid (14,15) from postoperative patients. Decreased levels of anti-inflammatory markers like Nrf2 (16) and IL-1ra (12) indicate that there is an imbalance between pro-inflammatory and anti-inflammatory activity in elderly hip fracture patients. Preclinical studies in animal models support these findings, showing that increased cytokine expression in rodent hippocampi after surgery correlates with cognitive decline (17). Key mediators of this postoperative neuroinflammatory response include IL-1β, IL-6, and TNF-α (18,19).

Additionally, disruptions in brain energy metabolism have been linked to neurodegenerative diseases (20). Nicotinamide adenine dinucleotide (NAD+) is crucial for cellular energy metabolism and serves as a key substrate for enzymes like poly-ADP-ribose polymerase (PARP), sirtuins, and cyclic ADP-ribose hydrolase (CD38), which regulate DNA repair, cell longevity, and immune responses (21). Given the age-related decline in NAD+ levels (21), there is growing interest in NAD+ precursors as potential neuroprotective therapies (22,23).

Nicotinamide (NAM), the soluble form of vitamin B3, is a key precursor for NAD+ synthesis via the salvage pathways (22). Thus, NAM supplementation supports NAD+ levels and offers neuroprotection through mechanisms such as anti-inflammatory effects (24) and stimulation of neuroprotective macrophages (25).

Our study aims to investigate the potential protective role of NAM supplementation in an animal model of bone fracture-induced cognitive impairment, to determine its impact on synaptic plasticity, inflammation, and memory.

## Materials and Methods

### Primers and Antibodies

A detailed list of the primers and antibodies used, and a detailed description of the quantitative polymerase chain reaction (qPCR) protocols, and Western blot analysis used in this work, is provided in Supplementary Appendix, Materials and Methods.

### Animals

All procedures followed the American Physiological Society’s guidelines for Research Involving Animals and were approved by the Universidad de Chile’s Bioethics Committee (approval number 19321-MED-UCH, CBA # 1111 FMUCH). Adult female C57BL/6 wild-type (WT) mice, aged 20–22 months, were used. Mice had ad libitum access to food and water and were kept at 21 ± 1°C, 55% humidity, with a 12-hour light/dark cycle (08:00 to 20:00). Female mice were selected for all experiments to avoid bias because of the reported sex differences in electrophysiological and cognitive responses (26,27)

### Traumatic Bone Injury Model

To simulate human long-bone fractures, we used a traumatic bone injury model described by Xiong et al. (28), which involves intramedullary pinning and tibial fracture under general anesthesia and analgesia. An aseptic open tibial fracture with intramedullary fixation was performed under isoflurane and buprenorphine anesthesia. The wound was irrigated and sutured. Post-surgery, animals recovered from anesthesia; analgesia (buprenorphine, 0.1 mg/kg) was given post-induction and pre-incision.

The sham animals were subjected to anesthesia and received a skin incision (similar to the surgical model), knee visualization, and then a skin suture. After that, the analgesic buprenorphine was administered postoperatively. The animals were allowed to recover spontaneously from the anesthetic. Thus, the sham and surgery groups underwent the same intervention process, the only difference being the fracture.

### NAM Administration

NAM (300 mg/kg; Sigma, St. Louis, MO) or saline (NaCl 0.9%) was administered intraperitoneally daily, in a volume of 0.1 mL, starting 5 days before bone trauma surgery and continuing for 3 days post-surgery.

### Open Field Test

The open field test (OFT) was performed as described by Peng et al. (29). Mice were placed for 5 minutes in the center of a 40 × 40 × 40 cm open field chamber. Their movements were tracked using a zenithal video camera and animal tracking software. The parameters recorded included total distance covered, time spent in the center, freezing time, and latency to reach the center. The floor was cleaned with 70% ethanol between tests.

### Y-Maze Test

The Y-maze test (YMT), performed as described by Peng et al. (29), employed a gray polyvinylene maze with 3 arms set at 120-degree angles. The experiment comprised 2 trials spaced 2 hours apart. During the initial trial, mice explored the start arm and one other arm, while the novel arm remained inaccessible. In the subsequent trial, all arms were accessible for a 5-minute period. Spatial recognition memory was evaluated by tracking arm entries and time spent in each arm. Spontaneous alternation, an indicator of exploratory behavior, was calculated using the formula described by Miedel et al. (30).


$$\begin{array}{lll} & Spontaneous\;alternation\;\%=\; & \frac{Total\;number\;of\;spontaneous\;alternations}{Total\;number\;of\;arm\;entries-2}\;\times \;100 \end{array}$$

The number of alternations is defined as successive entries into 3 different arms, forming overlapping triplet sets. For example, entries into arms 1, 3, and 2 are considered an alternation. In contrast, entries into arms 1, 2, and 1 would not be considered an alternation (30).

### Hippocampal Slice Preparation

Brains were removed by decapitation under isoflurane deep anesthesia 72 hours post-surgery. The hippocampus was dissected in cold buffer (in mM: 212.7 sucrose, 5 KCl, 2 MgCl_2_, 1 CaCl_2_, 10 glucose, 1.25 NaH_2_PO_4_, 26 NaHCO_3_, bubbled with 95% O_2_/5% CO_2_, pH 7.4), sliced to 300 μm using a vibratome, and stored in artificial cerebrospinal fluid (ACSF) containing (in mM: 124 NaCl, 5 KCl, 1.25 NaH_2_PO_4_, 1 MgCl_2_, 2 CaCl_2_, 10 glucose, 26 NaHCO_3_, pH 7.4), in 95% O_2_/5% CO_2_, for 2 hours at 30 ± 2°C prior to electrophysiological determinations.

### Electrophysiological Determinations

Experiments were conducted according to Arias-Cavieres et al. (31). Hippocampal slices were superfused with ACSF (95% O_2_/5% CO_2_) at 30 ± 2°C. Field excitatory postsynaptic potentials (fEPSPs) were evoked by square current pulses (0.2 ms) via a concentric bipolar stimulating electrode placed in the Schaeffer collateral–commissural fibers and were recorded in the CA1 *stratum radiatum* region using glass microelectrodes filled with ACSF. Basal synaptic transmission was assessed by applying pulses of 25–150 μA to generate an input/output (I-O) curve. Paired-pulse facilitation (PPF) was evaluated by applying 2 pulses every 15 seconds with interstimulus intervals from 20 to 200 ms. For long-term potentiation (LTP) evaluation, fEPSP responses were induced by applying stimulus adjusted to the half-maximal response displayed by the I-O curves; pulses were applied every 15 seconds until a stable baseline was recorded for 15 minutes. LTP was induced using the theta-burst stimulation (TBS) protocol, which consisted of 4 trains delivered every 20 seconds. Each train included 10 bursts at 5 Hz, with each burst comprising 4 pulses at 100 Hz.

The fEPSP recordings were continued for 60 minutes post-TBS, filtered at 10 kHz, and digitized at 5 kHz using Igor Pro.

### Golgi Staining

Dendritic spines were detected using the FD Rapid GolgiStain Kit (FD NeuroTechnologies, Columbia, MD) following manufacturer guidelines. Coronal slices (150 μm thick) were obtained with a vibratome, and images were captured with a 60× objective. At least 3 slices and 6 neurites per slice were analyzed. Neurites had to belong to a neuron with a visible soma, be in the CA1 zone of the hippocampus, and had 20–50 μm in length. Spine density was determined per 10 μm; spines were classified according to Risher et al. (32) using the Dendritic Spine Counter ImageJ plugin (33).

### Reverse Transcription-PCR

Total RNA from hippocampal tissue was isolated using Trizol reagent; RNA purity was checked by the 260/280 absorbance ratio, and cDNA was synthesized from 2 μg of total RNA using the High-Capacity cDNA Reverse Transcription Kit (Applied Biosystems). Real-time qPCR was performed on an AriaMx Real-Time PCR System (Agilent Technologies) system with SYBR Green dye, using specific primers for PARP-1, SIRT1, actin, IL-6, IL-1β, and TNF-α. The levels of mRNA were normalized to actin mRNA and were quantified using the 2−ΔΔCt method (34). Dissociation curves were analyzed to confirm product purity, and all samples were run in triplicate.

### Western blot analysis

Homogenized hippocampal extracts were resolved by 10% sodium dodecyl sulfate–polyacrylamide gel electrophoresis (SDS-PAGE) and transferred to polyvinylidene difluoride (PVDF) membranes. Blots were blocked in TBS with 0.2% Tween-20 and 5% fat-free milk, followed by overnight incubation at 4°C with primary antibodies: PARP-1 (1:1 000), Sirt1 (1:1 000), CD38 (1:1 000), brain-derived neurotrophic factor (BDNF; 1:4 000), and ryanodine receptor (RyR; 1:800). Membranes were then incubated with horseradish peroxidase (HRP)-conjugated secondary antibodies and developed using enhanced chemiluminescence. Membranes were stripped and re-probed with β-actin antibody (1:10 000) for loading correction. Band densities were analyzed using ImageJ software.

To detect RyR *S*-glutathionylation, hippocampal tissue was homogenized in nonreducing loading buffer containing urea and *N*-ethylmaleimide. Proteins were separated by SDS-PAGE under nonreducing conditions, transferred to PVDF membranes and incubated with anti-glutathione antibody (1:800) for 3 hours at room temperature, followed by secondary antibody incubation. Densitometric analysis was expressed as the ratio of anti-GSH/RyR band densities (35).

### Immunofluorescence

Brains were removed by decapitation under isoflurane deep anesthesia 72 hours post-surgery. After, the brains were removed and fixed by diffusion in 4% paraformaldehyde for 48 hours at room temperature. Subsequently, it was incubated for 72 hours at 4°C in a solution containing 30% sucrose, and 0.002% sodium azide for cryopreservation. Brains were cut in the coronal plane with a sliding frozen cryostat at −30°C, obtaining slices of 30 μM thickness. Free-floating sections were immersed for 2 hours at room temperature in phosphate-buffered saline (PBS) containing 0.25% Triton X-100 (PBS-TX) plus 3% donkey serum and were incubated overnight at 4°C with PBS-TX containing primary antibodies (IBA-1, WAKO, catalog N° 019-19741, 1:250 and NeuN, ABCAM, catalog N° 1042-24, 1:300). Coronal slices were washed 4 times for 5 minutes in PBS and then incubated for 2 hours with secondary fluorescent antibodies (Alexa Fluor 488 Donkey Anti-rabbit, #A-21206, Alexa Fluor 635 goat anti-mouse, #A-31574; Invitrogen). Nuclei were stained with 4′,6-diamidino-2-fenilindol (DAPI) (1:10 000, Sigma, MA). Brain tissue slices were washed in PBS, mounted on glass slides and covered with a mounting medium. A *z*-stack of 1.5 mm sections was captured from different hippocampal regions (CA1, CA3, and dentate gyrus [DG]), using a Nikon C2+ confocal microscope (Melville, NY), and fluorescence intensity was analyzed using NIS-Elements software viewer 4.0 and the open-source FIJI (ImageJ). All samples were prepared using a standardized protocol to ensure uniform staining and avoid variations in fluorescence intensity due to inconsistent treatment or handling. To further ensure that any observed differences in fluorescence were not artifacts of the imaging process, all images were captured under identical experimental conditions, with negative controls included and imaging performed in a blinded manner.

### Microglia Morphological Analysis

We analyzed microglial morphology using an image analysis tool developed by Martinez et al. (2023). Maximum intensity projections of the ionized calcium-binding adapter molecule 1 (Iba1; 488 nm) images were generated, segmented using ImageJ, and manually corrected. Full details and source code are available online (https://github.com/embl-cba/microglia-morphometry#microglia-morphometry). Image analysis was conducted blind to experimental conditions (36). The “New Microglia Segmentation and Tracking” plugin was utilized for cell segmentation, with parameters set to a minimum cell size of 200 μm and a maximum skeleton length of 450 μm. Shape and intensity features for each segmented cell were developed using the “Measure Microglia Morphometry” plugin. Morphological features were derived from these measured parameters.


$$\begin{array}{l} \begin{array}{ll} Solidity=\frac{Area}{Convex\;area};\; & Geodesic\;diameter=\frac{Geodesic\;diameter^{2}}{Area}\; \end{array} \end{array}$$

Specifically, solidity is linked to round, ameboid morphology, while geodesic elongation correlates with hypertrophic microglia (36).

### Measurement of Intracellular NAD+ Levels

The NAD+ concentration was determined using an NAD+/**nicotinamide adenine dinucleotide (NAD) + hydrogen (H)** (NADH) assay kit (MET-5014; Cell Biolabs, Inc., St. Louis, MO), following the manufacturer’s instructions. Briefly, hippocampus samples (100 mg) were homogenized in 500 µL of extraction buffer, sonicated, and centrifuged. To isolate NAD+, samples were treated with NaOH at 80°C, then neutralized. Absorbance readings were taken after incubation with NAD+ cycling reagent using a spectrophotometric reader at 450 nm.

### Statistical Analyses

Statistical analyses were performed using Prism 5 (GraphPad Software, Inc.). Values represent mean ± SEM. The Shapiro–Wilk normality test was applied. For multiple groups, 2-way analysis of variance, followed by Holm–Sidak’s post hoc test or Kruskal–Wallis multiple comparison test, was used; *p* < .05 was considered statistically significant. Further details are provided in figure legends.

## Results

### Treatment With NAM Prevents the Decline in Hippocampal Synaptic Plasticity Following Tibial Fracture Surgery

We conducted a comprehensive assessment of synaptic plasticity, focusing on LTP in the Schaffer collateral–CA1 pathway, following a previous report of fracture-associated synaptic disruptions in mice (28).

**Figure 1.**
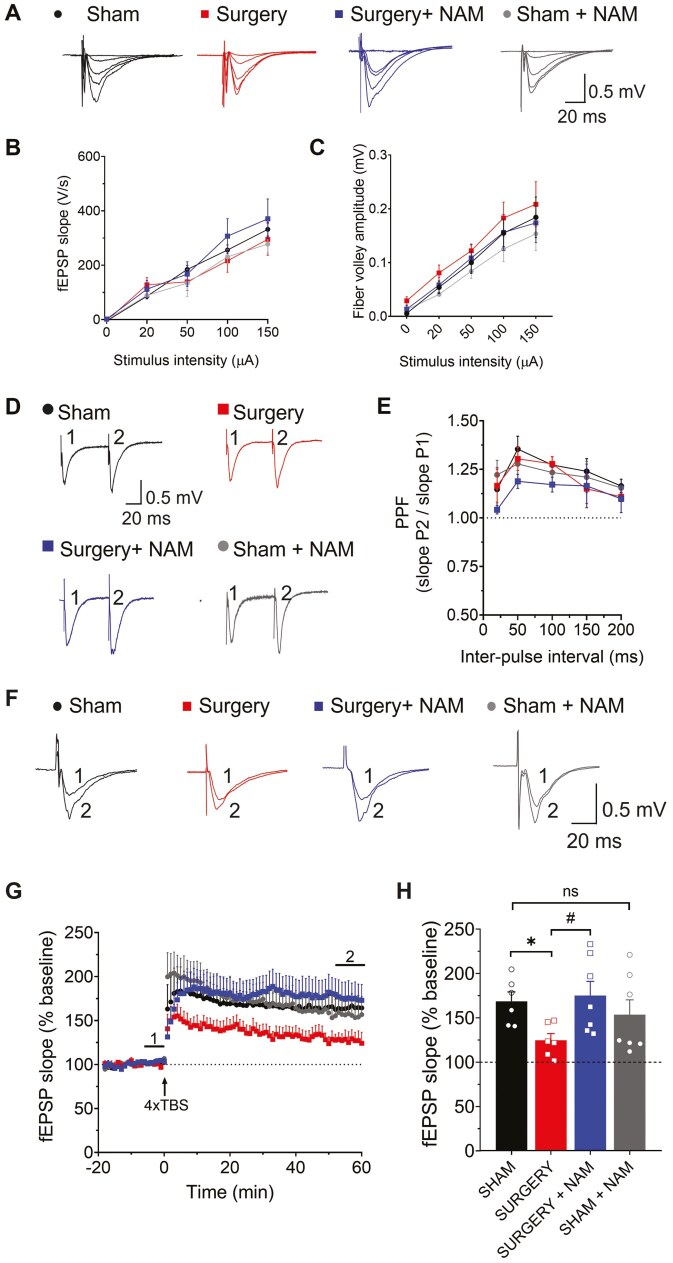
Nicotinamide (NAM) prevents surgery-induced synaptic plasticity deficits in excitatory hippocampal synapses. (A) Representative fEPSP traces from hippocampal slices in the following groups: Sham, Sham + NAM, Surgery, and Surgery + NAM. (B) fEPSP slope versus stimulus intensity. (C) Fiber volley amplitude versus stimulus intensity. (D) Paired-pulse facilitation responses. (E) Representative fEPSP traces at 50 ms interstimulus intervals. Data are mean ± SEM; (8, 4), with the first number indicating hippocampal slices and the second animals used. Two-way ANOVA, *p* > .05. (F) Representative fEPSP traces 1–5 minutes before (trace 1) and 60 minutes after TBS (trace 2) from the same groups. (G) Time course of fEPSP slopes (Schaffer collateral–CA1) before and after TBS. Group sizes: Sham (11, 6), Sham + NAM (13, 7), Surgery (10, 6), Surgery + NAM (13, 7). (H) Average fEPSP slopes during the last 10 minutes after TBS. Surgery group had lower fEPSP slopes (*p = .044) compared to Sham. Surgery + NAM group had higher fEPSP slopes (^#^*p* = .036) compared to Surgery. Data are mean ± SEM. Two-way ANOVA followed by Holm–Sidak’s post hoc test. ANOVA = analysis of variance; fEPSP = field excitatory postsynaptic potential; TBS = theta-burst stimulation.

Initially, we assessed fEPSP responses using I-O curves, measuring fEPSP strength at increasing stimulus intensities in hippocampal slices from 4 groups of mice: Sham, Surgery, Sham + NAM, Surgery + NAM. We found no differences in fEPSP slopes or fiber volley amplitudes between these 4 groups, indicating preserved baseline excitability (Figure 1A–C).

Next, we evaluated the PPF response, which reflects presynaptic activity (37). All 4 groups displayed similar and consistent responses to the second stimulus across all recordings, indicating comparable presynaptic function among groups (Figure 1D and E).

Following baseline assessment, LTP was induced using TBS (Figure 1F–H). Sham slices showed an increase in fEPSP initial slope to 168.7 ± 10.6% of baseline 1-hour post-stimulation, indicating successful LTP induction (Figure 1G). Conversely, Surgery slices exhibited reduced LTP levels (124.7 ± 7.5%) compared to slices from the sham mice group (Figure 1H; *p = .044), consistent with previous findings (28). Notably, NAM pretreatment preserved LTP levels (177.3 ± 18.7%) in the Surgery group, which were similar to those displayed by the Sham and the Sham + NAM groups, and significantly higher than those displayed by hippocampal slices from Surgery mice (^#^*p* = .036).

These findings highlight significant surgery-induced impairment in hippocampal synaptic plasticity 72 hours post-surgery. Of note, NAM pretreatment effectively preserved synaptic plasticity, indicating its protective role against surgery-induced synaptic plasticity dysfunction.

### Treatment With NAM Prevents the Deficits Induced by Surgery in RyR Calcium Channels (RyR2) Oxidation, Preserves Spine Density, and Promotes BDNF Levels

**Figure 2.**
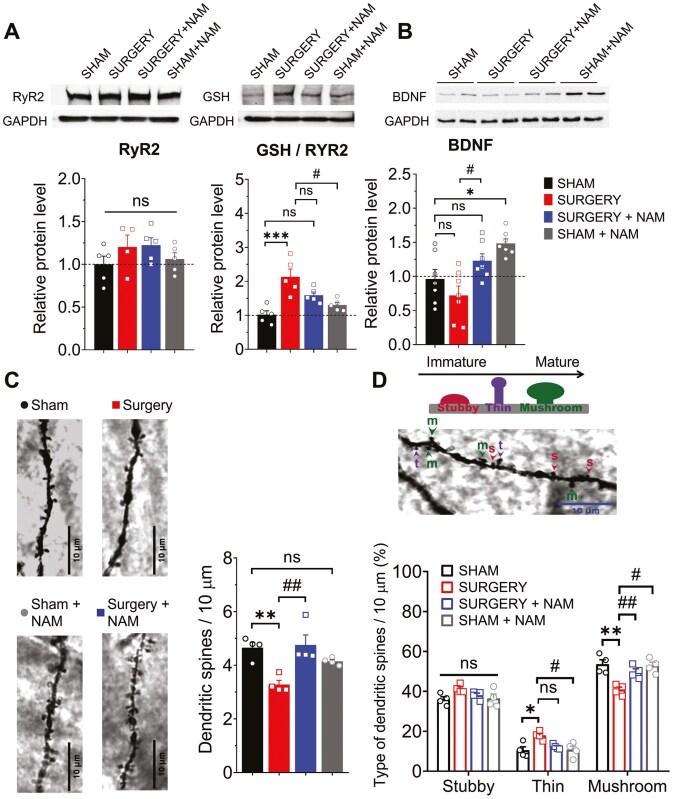
Effects of nicotinamide (NAM) treatment on hippocampal synaptic plasticity markers and dendritic spine density following surgery. (A) Representative blots of RyR2 expression (left) and RyR2 *S*-glutathionylation levels (right) in CA3–CA1 hippocampal homogenates from mice, obtained 72 hours post-surgery from the Sham, Sham + NAM, Surgery, and Surgery + NAM groups. Below, Western blot analysis levels normalized to total GAPDH levels. Enhanced RyR2 *S*-glutathionylation was observed in slices from the Surgery group (***p < .001) compared to slices from the Sham group. However, slices from the Surgery + NAM and the Sham + NAM groups showed partial attenuation of this effect; *n* = 5 animals per group. (B) Representative blots of BDNF expression in CA3–CA1 hippocampal homogenates from the 4 mentioned groups are depicted. Below, Western blot analysis levels normalized to total GAPDH levels. NAM administration significantly elevated BDNF levels after surgery compared to mice exposed to surgery without NAM treatment (^#^*p* = .036); *n* = 7 animals per group. (C) Representative images of neurites from the CA1 region of mouse hippocampal slices stained with FD rapid Golgi Stain are depicted for the 4 mentioned groups (left). The scale bar represents 10 µm. Right, spine density analysis was performed by calculating the ratio between the number of spines and the dendritic length (in 10 μm). The density of dendritic spines showed a significant decrease in the total number of spines following surgery (**p < .01). This decrease was prevented by NAM treatment (^##^*p* < .01). (D) Graphical representation of the percentage of spines as mushroom-like, thin, and stubby-shaped per total spines of apical dendrites in the CA1 subarea. The surgery group displayed a significant increase in thin-shaped spines (*p < .019), accompanied by a decrease in mushroom-like spines (**p < .01). This latter change was prevented by NAM treatment (^##^*p* < .01). A total of *N* = 60 dendrites from 4 animals per group were analyzed. Values represent mean ± SEM. Statistical significance was assessed with 2-way ANOVA followed by Holm–Sidak’s post hoc test. ANOVA = analysis of variance; BDNF = brain-derived neurotrophic factor.

LTP requires the intracellular calcium signals mediated by the endoplasmic reticulum resident RyR channels (38). The RyR channels, known for their cysteine-rich structure, are considered cellular redox sensors (39) due to their susceptibility to oxidative modifications, which by promoting their activation by calcium, can lead to unregulated calcium leakage and disrupt synaptic transmission (40). Here, we investigated if tibial fracture and NAM supplementation influence the hippocampal content and the oxidation (*S*-glutathionylation) levels of the type-2 (RyR2) isoform (Figure 2). We found that neither surgery nor NAM treatment affected RyR2 protein levels (Figure 2A). However, surgery significantly increased RyR2 *S*-glutathionylation levels (***p < .001), an indication that surgery increased the hippocampal oxidative tone. NAM administration appeared to partially mitigate this increase, showing a trend (*p* = .06) that did not reach statistical significance.

Alongside calcium signals, BDNF and dendritic spine remodeling play crucial roles in hippocampal synaptic plasticity and spatial memory (38). We investigated whether the protection afforded by NAM against the noxious effects of surgery modifies BDNF expression and dendritic spine density.

We found that surgery induced a slight decrease in BDNF levels, and that NAM significantly elevated hippocampal BDNF expression compared to the BDNF levels displayed by hippocampal samples from untreated surgery mice (Figure 2B; ^#^*p* = .036). Additionally, using Golgi–Cox staining, we observed a significant reduction in total dendritic spine density post-surgery (Figure 2C; **p < .01). Of note, NAM pretreatment preserved spine density to levels comparable to those displayed by samples from sham-operated mice (Figure 2C; ^##^*p* < .01). Additionally, surgery led to reduced mushroom-like spines and increased thin spines (Figure 2D, **p < .01; *p < .01), indicating a shift toward more immature spine forms (32,41). Remarkably, NAM pretreatment preserved mushroom-like spine density in the Surgery + NAM group, with values similar to those displayed by samples from sham-operated mice and significantly higher than those displayed by samples from the Surgery group (^##^*p* < .01).

These results show that the surgery-induced loss of dendritic spines primarily decreases mature spine density, while NAM preserves spine density and mature type in the Surgery + NAM group. Overall, these findings underscore the potential protective role of NAM against surgery-induced synaptic plasticity changes and enhanced oxidation.

### Treatment With NAM Prevents Tibial Fracture Surgery-Induced Hippocampal-Related Cognitive Decline

**Figure 3.**
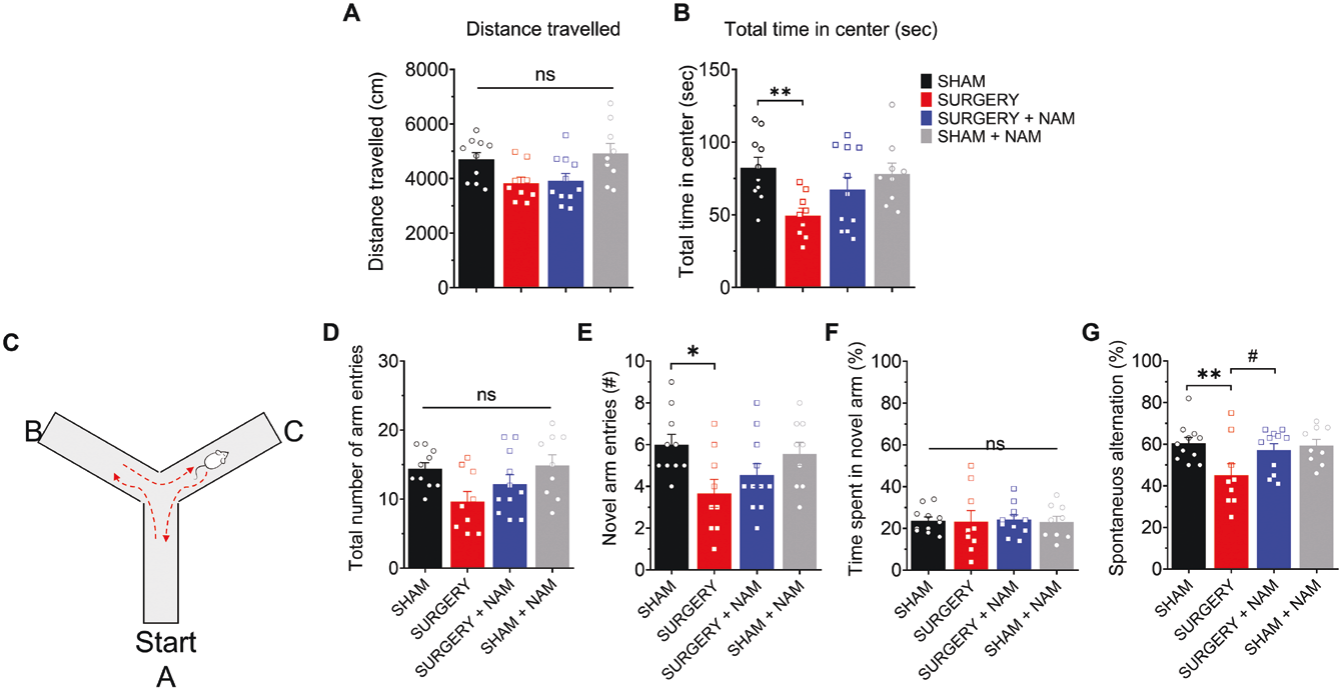
After surgery, mice displayed impaired natural (open field test) and learned (Y-maze test) behaviors, measured 72 hours post-surgery; NAM administration prevented these changes. (A) Open field test. At 72 hours post-surgery, there were no significant differences in locomotor activity between Surgery and Sham groups. All groups traveled a similar distance during the exploration time. (B) When assessing the time spent exploring the center of the maze, the Surgery group (*n* = 9) exhibited a significant decrease compared to the Sham condition (*n* = 10; **p = .006). This deficit was not observed in the Surgery group that received preventive treatment with NAM (*n* = 11). (C) Illustrations of the Y-maze of the alternations based on the sequence of arm visits. (D) Y-maze test: The surgery did not significantly affect the total number of arm visits made by the mice compared to the sham condition. However, it significantly altered the number of visits to novel arms (E) without changing the percentage of time spent exploring the new arm (F). (G) When assessing the spontaneous alternation behavior, the Surgery group (*n* = 9) exhibited a significant decrease compared to the Sham condition (**p = .002). All these effects were prevented by NAM pretreatment (*n* = 11; ^#^*p* = .01). Values represent mean ± SEM. Sham condition (*n* = 10), Surgery group (*n* = 9), Surgery + NAM group (*n* = 11), and Sham + NAM group (*n* = 9). Statistical significance was assessed with 2-way ANOVA followed by Holm–Sidak’s post hoc test. ANOVA = analysis of variance; NAM = nicotinamide.

Previous studies have shown that orthopedic surgery induces attention deficits in a mouse model resembling POD (17). In our study, we initially assessed locomotor activity, anxiety-like behavior, and hippocampal-dependent memory using the OFT and YMT, respectively (Figure 3). The OFT revealed no significant differences in locomotor activity between groups, measured by the total distance traveled (Figure 3A; *p* = .22), indicating preserved motor function in the mice after surgery. However, there was a notable decrease in the total time spent in the center zone post-surgery (Figure 3B; **p = .006), suggestive of potential anxiety-like behavior. Interestingly, mice pretreated with NAM spent a similar amount of time exploring the center zone as the sham-operated group (Figure 3B; *p* = .09), suggesting a mitigating effect of NAM on surgery-induced anxiety-like behavior in the OFT, despite no significant impact on overall locomotor activity.

Subsequently, to evaluate learning and memory, 1 hour after the OFT we conducted the Y-maze spontaneous alternation test. Total arm entries did not differ significantly among groups, indicating no motor deficits (Figure 3D; surgery vs sham, *p* = .08). However, surgery reduced the novel arm entries (Figure 3E; *p = .01) and spontaneous alternation rates (Figure 3G; **p < .01) compared to those displayed by sham-operated mice. This impairment was prevented in the surgery group pretreated with NAM (Figure 3G, ^#^*p* = .01), which displayed a behavior resembling that of the sham-operated group. Overall, these findings indicate that NAM pretreatment mitigates cognitive impairments, and the anxiety-like behavior induced by tibial fracture surgery in mice.

### Treatment With NAM Regulates Neuroinflammation in the Hippocampus of Mice Following Tibial Fracture Surgery

**Figure 4.**
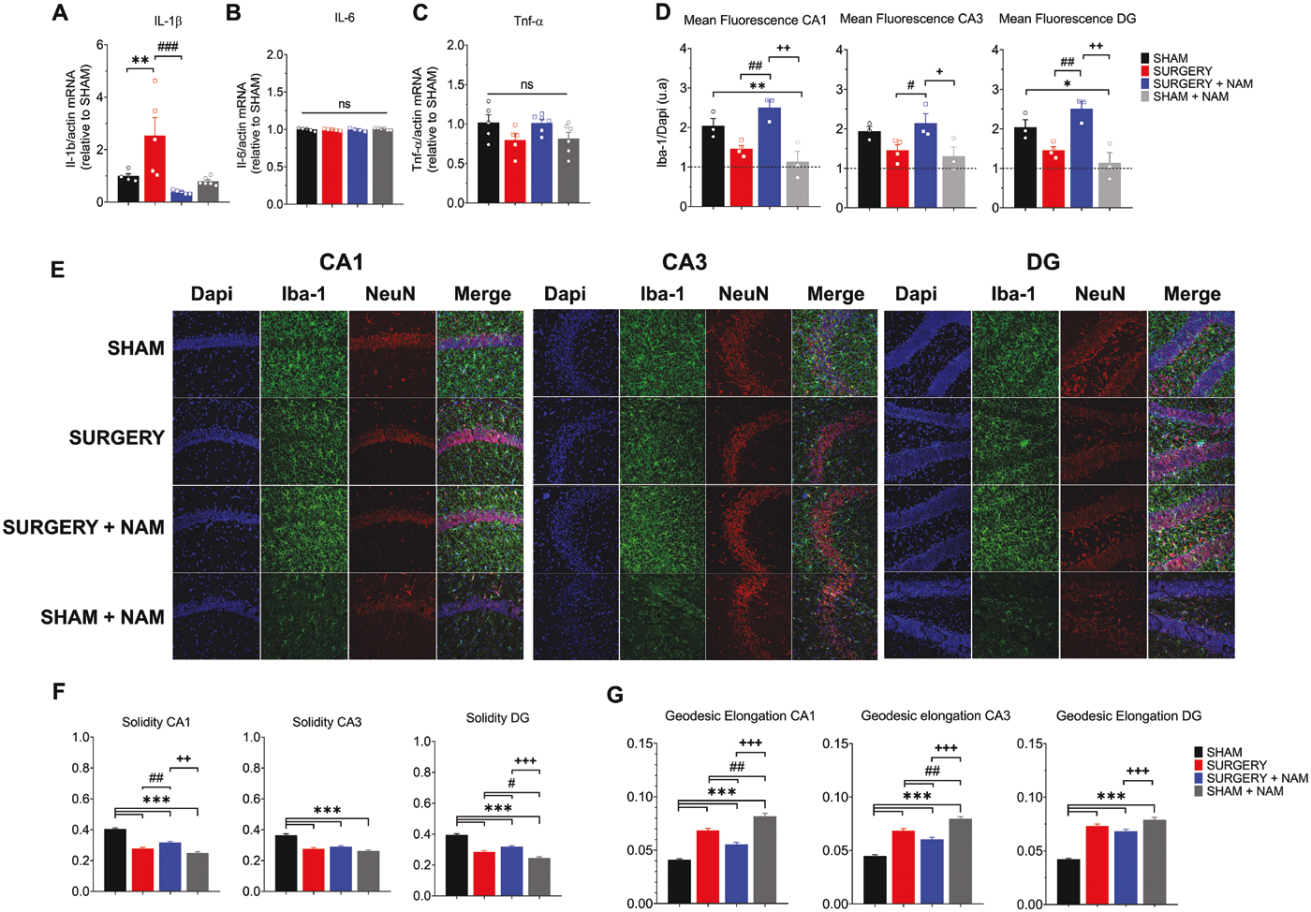
Nicotinamide (NAM) administration prevents surgery-induced inflammation in the mouse hippocampus. (A) RT-PCR quantification in the Sham, Sham + NAM, Surgery, and Surgery + NAM groups. At 72 hours after surgery, hippocampal IL-1β levels were significantly increased in the Surgery group compared to the Sham group (**p < .01). However, the Surgery group treated with NAM showed a significant decrease in IL-1β mRNA levels compared to the Surgery group (^###^*p* < .001). (B, C) No significant differences were observed in IL-6 (B) or TNF-α (C) levels among the 4 groups. (D) Mean fluorescence quantification for the 4 groups across the 3 hippocampal sections. (E) Immunofluorescence staining for the Iba1 marker in the CA1, CA3, and dentate gyrus (DG) regions of hippocampal sections from the 4 groups. (F, G) Morphological analysis of microglia segmentation. Values represent mean ± SEM; (A–C) *n* = 5–6; (D–G) *n* = 3–4 animals per group. Statistical significance was assessed using 2-way ANOVA followed by Holm–Sidak’s post hoc test (A–E) and Kruskal–Wallis nonparametric test (F, G). *p < .05; **p <.001; ***p < .001. ANOVA = analysis of variance; RT-PCR = reverse transcription-polymerase chain reaction.

Several studies underscore the pivotal role of neuroinflammation in POD. In particular, Cibelli et al. (42) used C57BL/6J knockout (IL-1 receptor-deficient) and WT mice to show that fracture increases hippocampal IL-1β levels and microgliosis. Accordingly, following surgery, we quantified the hippocampal mRNA levels of IL-1β, IL-6, and TNF-α using quantitative reverse transcription-PCR (RT-PCR; Figure 4). Increased levels of IL-6 and TNF-α indicate inflammation and typically increase together with IL-1β (18). The IL-1β mRNA levels were significantly higher 72 hours post-surgery compared to the sham group (Figure 4A; **p < .01). However, mice that underwent surgery and received preventive NAM treatment showed significantly lower IL-1β mRNA levels compared to the surgery group (Figure 4A; ^###^*p* < .001). In contrast, IL-6 and TNF-α levels remained unchanged between groups (Figure 4B and C), suggesting that the surgery-induced deficits in synaptic plasticity and cognition may primarily relate to IL-1β-mediated inflammatory responses.

To evaluate the hippocampal microglial response to tibial fracture surgery and NAM supplementation, we used confocal microscopy to analyze microglial features by immunocytochemistry, quantitatively using the Iba1 as a cytoplasmic marker for microglia. Quantification of Iba1 mean fluorescence intensity revealed group differences. Notably, in the CA1, CA3, and DG hippocampal sections from mice exposed to tibial fracture surgery, we observed only small and mostly insignificant changes in Iba1 levels. However, NAM treatment induced significant changes in Iba1 levels compared to sections from the surgery group (Figure 4D; ^##^*p* < .01 for CA1 and DG; ^#^*p* = .049 for CA3 hippocampal sections). NAM pretreatment appeared to increase Iba1 levels in the surgery group, while it had no effect in the sham group. Under sham conditions, Iba1-labeled microglial cells showed a uniform distribution across CA1, CA3, and DG hippocampal sections, characterized by a mosaic-like pattern with small soma and ramified processes (Figure 4E).

To further analyze microglial adaptations, we segmented individual Iba1+ cells and assessed morphological parameters using a publicly available image analysis tool (36). This analysis revealed significant changes (Figure 4F and G); in CA1, CA3, and DG hippocampal sections both surgery and NAM-treated mice showed reduced solidity parameters (indicative of round or ameboid-like morphology) compared to sham conditions (Figure 4F). Conversely, geodesic diameter (associated with hypertrophic and elongated processes) was significantly increased in surgery and NAM-treated mice compared to sham conditions across all hippocampal sections (Figure 4G). These results indicate that both surgery-induced inflammation and NAM treatment induce phenotypic changes in microglia from a basal to a reactive state. Overall, these findings highlight the role of NAM in modulating the neuroinflammatory responses linked to IL-1β and modulating microglial activity following surgery.

### Surgery Alters the Levels of NAD+-Consuming Enzymes in the Hippocampus

NAM is a crucial precursor in NAD+ biosynthesis via the salvage pathway (21). Inflammatory processes influence the expression of key enzymes involved in NAD+ availability (43–45). Therefore, we examined the expression of 3 pivotal enzymes in NAD+ metabolism: CD38, PARP-1, and SIRT1 (21), and hippocampal NAD+ levels.

**Figure 5.**
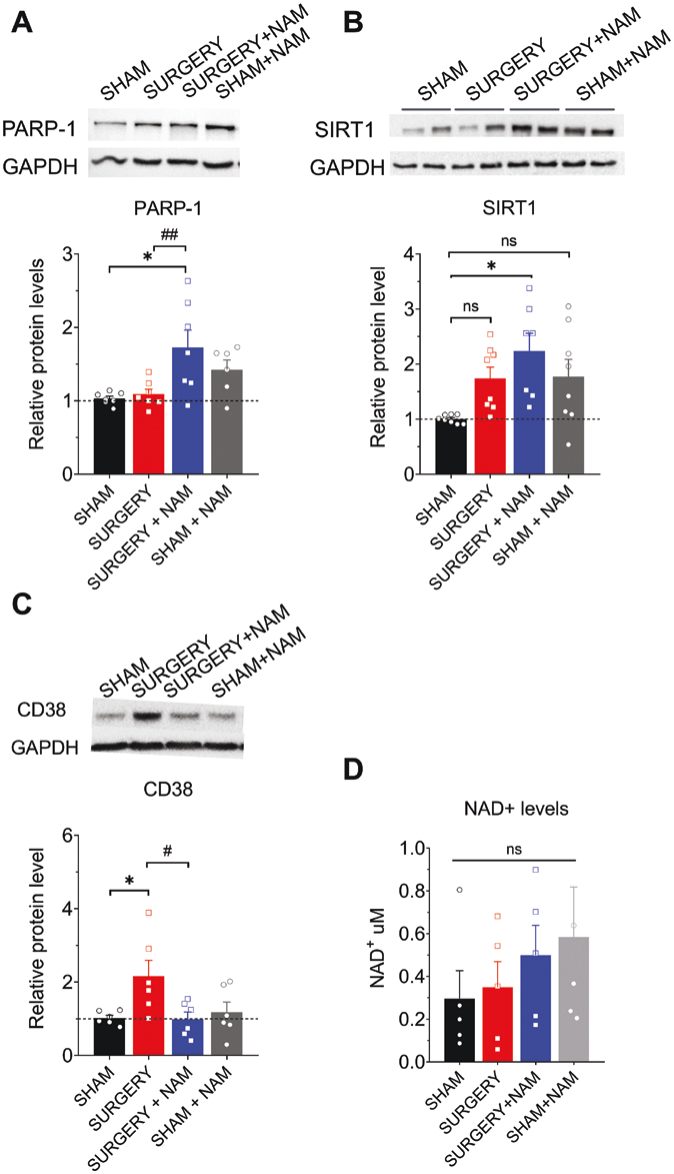
Effects of NAM on hippocampal NAD+-consuming enzymes and NAD+ cofactor availability following surgery. Representative blots of PARP-1 (A), SIRT1 (B), and CD38 (C) protein contents in CA3–CA1 hippocampal homogenates from mice, measured 72 hours post-surgery in the Sham, Sham + NAM, Surgery, and Surgery + NAM groups. Western blot analysis levels were normalized to total GAPDH levels. (A) Representative blots of PARP-1 expression. NAM administration significantly elevated PARP-1 levels in the samples from the Surgery + NAM group compared to the Sham group (*p = .01) and the Surgery group (^##^*p* = .007); *n* = 6–7 animals per group. (B) Representative blots of SIRT1 protein content in hippocampal samples from the 4 groups. NAM administration significantly elevated SIRT1 levels in the Surgery group compared to the Sham group (*p = .01); *n* = 7–8 animals per group. (C) Markedly increased CD38 expression was observed in slices from the Surgery group (*p < .017) compared to slices from the Sham group. Slices from the Surgery + NAM group showed an attenuation of this effect (^#^*p* = .014), while Sham + NAM group did not have changes; *n* = 5–6 animals per group. (D) Quantification of hippocampal NAD+ levels measured by spectrophotometry revealed no significant decline in the Surgery group compared to the Sham group. Preventive NAM treatment promoted slight increases in NAD+ levels. Values represent mean ± SEM. Statistical significance was assessed with 2-way ANOVA followed by Holm–Sidak’s post hoc test. ANOVA = analysis of variance; NAM = nicotinamide; PARP = poly-ADP-ribose polymerase.

While surgery did not significantly alter PARP-1 and SIRT1 protein levels (Figure 5), pretreatment with NAM positively influenced their expression, resulting in significant increases compared to the sham group (Figure 5A and B; PARP-1, *p = .01, and SIRT1, *p = .01, respectively). Conversely, surgery markedly elevated CD38 protein levels compared to the sham group (Figure 5C; *p = .017), an effect notably attenuated by NAM treatment (^#^*p* = .014).

Finally, using spectrophotometric methods (Figure 5D), we evaluated the impact of surgery and NAM on NAD+ levels. We did not observe significant decreases in hippocampal NAD+ levels in mice after surgery. However, mice receiving preventive NAM treatment showed slight increases in NAD+ levels, although these changes were not statistically significant. Based on these findings, we suggest that the detrimental effects of surgery on cognition and synaptic plasticity may involve alterations in NAD+ metabolism rather than a decrease in its levels.

## Discussion

In the present study, we aimed to investigate the impact of NAM on the defects in synaptic plasticity and cognition, and the neuroinflammation induced by tibial fracture surgery in mice, as a plausible model of POD. Our results show that pretreatment with NAM mitigated surgery-induced deficits in neuronal plasticity and cognition, suggesting its potential as a therapeutic intervention for human POD.

Our electrophysiological findings highlight significant differences in synaptic plasticity among the groups. Specifically, the surgery group showed reduced LTP in hippocampal slices, indicating impaired synaptic plasticity function (46). However, this decline was markedly attenuated in the surgery group pretreated with NAM, suggesting a protective role of NAM in preserving synaptic plasticity function post-surgery. The observed increases in RyR2 oxidation induced by surgery suggest alterations in hippocampal redox state toward oxidation, which, by causing anomalous calcium signaling (40), could potentially underlie the electrophysiological alterations following fracture. Interestingly, these oxidative changes were mitigated by NAM pretreatment. In addition, the decrease in the expression of BDNF, a neurotrophic factor known as a crucial regulator of dendritic structural plasticity (38), may underlie the observed changes in dendritic spine density caused by surgery. Furthermore, NAM plays a pivotal role in modulating NAD+ metabolism, which is essential for neuronal function and plasticity (21). Specifically, NAM pretreatment resulted in increased levels of SIRT1 and PARP-1, 2 enzymes involved in NAD+ metabolism (22), potentially enhancing synaptic resilience to surgical stress.

Behavioral assessments using the Y-maze and OFT offered insights into the cognitive and anxiety-related protective impacts of NAM. Surgery notably impaired spontaneous alternation behavior in the Y-maze, reflecting deficits in spatial working memory. However, NAM pretreatment effectively prevented these deficits, highlighting its ability to preserve cognitive function in mice after surgery. Furthermore, the OFT demonstrated increased anxiety-like behavior in the surgery group, a response that was absent in the group pretreated with NAM. These findings raise the possibility that NAM could potentially alleviate surgery-induced anxiety, likely through its antioxidant, anti-inflammatory, and neuroprotective properties.

Neuroinflammation is a well-documented contributor to cognitive decline and POD (11). Following surgery, we observed a notable increase in IL-1β mRNA levels in the hippocampus, while TNF-α and IL-6 remained unchanged. Importantly, pretreatment with NAM significantly attenuated the surgery-promoted increase in IL-1β levels, suggesting that NAM has potent anti-inflammatory effects in the context of surgery-induced neuroinflammation. This NAM-mediated reduction in IL-1β levels could potentially mitigate the detrimental impact of neuroinflammation on synaptic plasticity and cognitive function (42). Additionally, the observed changes in microglial phenotype are worth noting. Specifically, surgery-induced inflammation and NAM treatment caused phenotypic alterations in microglia, increasing the proportion of hypertrophic or less ameboid-like forms, as evidenced by decreased solidity and increased geodesic elongation. While ameboid microglia are typically linked to inflammatory cytokine production, including IL-1β, and neurodegenerative conditions (47), recent evidence emphasizes the neuroprotective roles of ramified microglia in regulating neuronal and glial functions (48).

At 72 hours post-surgery, our data show no significant increase in microglial density or the Iba1/DAPI ratio compared to other groups. This may be attributed to transient microglial reactivity, which peaks shortly after surgery, as noted at 6 and 24 hours post-surgery (18,28,42). Notably, anti-inflammatory mediators such as IL-10 and IL-13 have been significantly increased postoperatively in both humans (49) and mice (50), indicating a widespread pro- and anti-inflammatory response. At the time of our evaluation, these anti-inflammatory mechanisms may have mitigated acute microglial activation, resulting in stable reactivity without changes in cell number or morphology, while still promoting IL-1β release.

Our results could not definitively determine whether the morphological changes induced by fracture surgery and/or NAM treatment reflect a neuroinflammatory or neuroprotective microglial response; it is likely that both processes are occurring simultaneously. This suggests that microglial activation spans a spectrum of phenotypes that may not always present as increased Iba1 expression but still significantly affect cytokine release and neuronal function. Further characterization, including metabolic and transcriptomic analyses, is needed to elucidate the specific roles of microglial subpopulations associated with neuroinflammatory and protective phenotypes. Another mechanism underlying NAM protective effects involves its influence on NAD+ metabolism. NAM enhances NAD+ bioavailability (22), which is essential for cellular energy metabolism and the function of NAD+-dependent enzymes, such as sirtuins and PARPs (45). The observed increase in SIRT1 and PARP-1 levels in NAM-pretreated mice supports this hypothesis. Moreover, the anti-inflammatory properties of NAM likely contribute to its protective effects by reducing IL-1β levels and altering microglial phenotype, potentially modulating the neuroinflammatory response underlying surgery-induced cognitive dysfunctions and POD.

Furthermore, the lack of significant changes in NAD+ levels between the groups suggests that NAM’s effects are not solely due to increases in NAD+ concentration but also involve modulation of NAD+ metabolism and related pathways. This distinction is crucial for understanding NAM’s nuanced role in neuroprotection.

Regarding the concerns about the potential effects of buprenorphine and anesthesia on hippocampal-dependent processes such as spatial memory and synaptic plasticity, these factors were shared by both groups. Therefore, any alterations in these parameters can be attributed to the fracture experience itself and not to the use of analgesic or anesthesia, as both groups were equally exposed to these conditions in a controlled manner.

The main strength of our study lies in the broad range of processes examined in the model, including electrophysiology, histomorphology, mRNA and protein expression, and behavior. Together, these elements build the case supporting the neuroprotective role of NAM supplementation on surgery-induced cognitive dysfunctions in older mice. However, the limitations of our study include the need for more sensitive methodologies, such as metabolomic analysis to accurately evaluate NAD+ levels. Nonetheless, our findings pave the way for future research to delve deeper into the mechanisms by which NAM supplementation exerts its protective effects in this model.

The present findings hold significant clinical implications. POD is a prevalent and serious complication among older surgical adults aged 65 years and older, often resulting in prolonged hospital stays, increased healthcare costs, and long-term cognitive decline. Current treatments primarily focus on symptomatic relief rather than prevention. NAM, with its dual action on modulating NAD+ metabolism and reducing neuroinflammation, presents a promising avenue for preventive therapy. Future clinical studies should investigate the efficacy of NAM supplementation in reducing both the occurrence and severity of POD in surgical patients.

In conclusion, our study demonstrates that NAM pretreatment can mitigate surgery-induced cognitive deficits, anxiety-like behavior, and neuroinflammation in a probable mouse model of human POD. These findings underscore NAM’s potential as a therapeutic intervention to safeguard cognitive function and promote recovery in surgical patients. By targeting the underlying mechanisms of neuroinflammation and NAD+ metabolism, NAM may provide a novel approach to improving outcomes for patients at risk of POD.

## Supplementary Material

glae303_suppl_Supplementary_Materials
