# Supplementary material for: Nicotinamide Prevents the Plasticity Impairments and the Cognitive Dysfunction Caused by Bone Fracture in Older Mice
Source: J Gerontol A Biol Sci Med Sci. 2025 Jan 17;80(7):glae303. doi: 10.1093/gerona/glae303 (PMC12363222; doi:10.1093/gerona/glae303)
Supplement: glae303_suppl_Supplementary_Materials [file glae303_suppl_supplementary_materials.pdf]

Materials and Methods

Traumatic bone injury model.

We used the traumatic bone injury model, described by Xiong et al. (28) that consists of intramedullary pinning and tibial fracture under general anesthesia and analgesia, and thus mimics the skin injury, muscle trauma, and bone repair associated with common long-bone fractures and repair in humans. Surgery consisted of an aseptic open tibial fracture with intramedullary fixation performed under general anesthesia with isoflurane and buprenorphine. Briefly, the left hind paw was shaved and disinfected. A median paw incision was then performed, followed by the insertion of a 0.38-mm pin in the tibial intramedullary canal. The periosteum was stripped, and osteotomy was performed. After producing the fracture, the wound was irrigated and the skin was sutured with 5-0 Vicryl sutures (Ethicon); thereafter, animals were allowed to recover spontaneously from the anesthetic. Analgesia (s.c., buprenorphine, 0.1 mg/kg) was administered after anesthetic induction and before skin incision.

Open field test (OFT)

The OFT was performed as described by Peng et al. (29). Specifically, the mouse was gently placed in the center of an open field chamber (40 x 40 x 40 centimeters) under dim light and was allowed to move freely for 5 minutes. The movement parameters of the mouse were monitored and analyzed via a zenithal video camera connected to an animal tracking system software. The total distance moved (centimeters), the time (seconds) spent in the center of the open field, the freezing time (seconds), and the latency (the time in seconds for the mice to reach the location at the first attempt) to the center of the open field was recorded and analyzed. The floor of the open field was cleaned with 70% ethanol solution between each test.

Y maze test.

The Y maze test was performed as described by Peng et al. (29). Specifically, the Y maze, made of gray polyvinylene, was placed in a quiet, illuminated room. Each maze consisted of three arms (8 X 30 X 15 centimeters, width X length X height), with an angle of 120 degrees between each arm. The three arms included the start arm, in which the mouse starts to explore (always open); the novel arm, blocked at the first trial, but opened at the second trial; and the other arm (always open). In the experiment, the start arm and the other arm were designed randomly to avoid spatial memory errors. The Y maze test consisted of 2 trials separated by an inter-trial interval (ITI). The first trial (training) was 10 minutes, which allowed the mouse to explore 2 arms (the start arm and the other arm) of the maze, with the novel arm being blocked. After 2 hours of ITI, the second trial (retention) was conducted. For the second trial, the mouse was placed back in the maze in the same start arm with free access to all 3 arms for 5 minutes. A video camera, that was linked to the Any-Maze animal tracking system software, was installed 60 centimeters above the chamber to monitor and analyze the number of entries and the time spent in each arm. The time spent in and entries into the novel arms indicated the spatial recognition memory (learned behavior). Each of the arms of the Y maze was cleaned with 70% ethanol solution between trials. Spontaneous alternations are a behavioral test for measuring exploratory behavior (30). This test is based on the willingness of rodents to explore a new environment. Normal rodents will prefer to experience a different arm of the maze than the one they visited on their previous entry. Many parts of the brain including the hippocampus, septum, basal forebrain, and prefrontal cortex are involved in this task (38).

Each arm of the maze is labeled as either arm A, B, or C. In each session, the animal is placed in arm B and allowed to explore the three arms for 5 minutes. The number of arm entries

and the number of alternations is scored live to calculate the percent alternation. The entry is considered when all four limbs are within the arm. Spontaneous alternation, a measure of exploratory behavior (30), was calculated with the following formula.

$$Spontaneous\ alternation\ \% = \frac{total\ number\ of\ spontaneous\ alternations}{total\ number\ of\ arm\ entries - 2}\ x\ 100$$

The number of alternations is defined as successive entries into three different arms, forming overlapping triplet sets. For example, entries into arms 1, 3, and 2 are considered an alternation. In contrast, entries into arms 1, 2, and 1 would not be considered an alternation (30).

Hippocampal slice preparation

Brains were removed by decapitation under isoflurane deep anesthesia 72 hours post-surgery. The hippocampus was dissected in cold dissection buffer containing (in mM: 212.7 sucrose, 5 KCl, 1 MgCl2, 2 CaCl2, 10 glucose, 1.25 NaH2PO4, 26 NaHCO3, bubbled with 95% O2/5% CO2, pH 7.4) and cut into 300 µm transverse slices using a vibratome (Vibratome 1000 plus, Ted Pella Inc., Redding, CA). Hippocampal slices were then transferred to an immersion storage chamber kept at room temperature in artificial cerebrospinal fluid (ACSF) containing (in mM: 124 NaCl, 5 KCl, 1.25 NaH2PO4, 1 MgCl2, 2 CaCl2, 10 glucose, 26 NaHCO3, pH 7.4), in 95% O2/5% CO2, for 2 hours at 32°C for electrophysiological determinations. Alternatively, slices were immediately frozen in liquid nitrogen and stored at –80°C for subsequent biochemical analysis.

Electrophysiological determinations

Experiments were conducted according to Arias-Cavieres et al. (31). Hippocampal slices were superfused with ACSF (in 95% O2/5% CO2) at a rate of 2 ml/min at 30 ± 2°C. Field excitatory postsynaptic potentials (fEPSP) were evoked by square current pulses (0.2 ms)

delivered with a concentric bipolar stimulating electrode (FHC Inc., Bowdoinham, ME) located in the Schaeffer collateral–commissural fibers; fEPSP was recorded using glass microelectrodes (2–3 MΩ) filled with ACSF placed into the stratum radiatum of the CA1 region. To evaluate basal excitatory synaptic transmission, pulses of 25, 50, 75, 100, or 150 microamperes were applied to generate an input/output (I / O) curve. Results are presented as stimulus intensity versus Fiber Volley (FV) amplitude or fEPSP slope. To evaluate presynaptic components, two pulses were applied every 15 s, with inter-stimulus intervals starting at 20 ms and ending at 200 ms, doubling the interval after each trial. The results are presented as the ratio between the initial fEPSP slopes evoked by the second stimulus over the first. After monitoring both basal synaptic transmission and pre-synaptic responses, we evaluated LTP adjusting fEPSP to half of the maximal evoked response. Pulses were applied every 15 seconds until a stable baseline was recorded for at least 15 min. To induce Long-term potentiation (LTP), we used the theta-burst stimulation (TBS) protocol, comprised of four trains of 10 bursts at 5 Hz each, where each burst comprised four pulses at 100 Hz. In all experiments, fEPSP recordings were continued for 60 min after applying the TBS protocol. Recordings were filtered at 10 kHz and were digitized at 5 kHz, using Igor Pro (WaveMetrics Inc., Lake Oswego, OR).

Golgi Staining

Dendritic spines were measured using the FD Rapid GolgiStain Kit according to the manufacturer's guidelines (FD NeuroTechnologies, Columbia, MD, USA). Briefly, the dissected mouse brains were immersed in Solution A/B for two weeks in dark conditions at room temperature. The brains were placed in Solution C for 24 h in the dark. Afterward, coronal slices 150 µm thick were obtained using a vibratome and mounted on gelatin-coated microscope slides with Solution C. The sections were allowed to dry naturally at RT, and

then placed in a mixture of Solution D/E for 10 min. Next, sections were rinsed twice in Milli-Q water for 4 min each time. Finally, the sections were dehydrated, cleared in xylene, and mounted using Permount mounting media (Sigma-Aldrich, Burlington, MA, USA). Images of the slices were acquired with a 60X objective. A minimum of three slices per condition were analyzed, and at least 10 neurites were analyzed for each slice. To be considered for analysis, each neurite had to belong to a neuron with a visible soma, reside in the CA1 zone of the hippocampus (postsynaptic), and the same plane; within this plane, the extension of the neurite had to be between 20–50 μm in length. The z-stacks were acquired with the Nikon Nis Elements software (Nikon Instruments Inc, Melville, NY), for subsequent blind analysis using the ImageJ program. Spine density was defined as the number of spines per 10 μm. Spine density was determined per 10 μm; spines were classified according to Risher et al. (32) using the Dendritic Spine Counter ImageJ plugin (33).

RT-PCR.

Total RNA from hippocampal homogenized tissue was isolated using Trizol reagent. To remove any contaminating genomic DNA, a DNAase digestion step with TURBO DNA- free TM Kit was included. RNA purity was assessed by the 260/280 absorbance ratio. cDNA was synthesized from synthesized from 2 ug of total RNA using the High-Capacity cDNA Reverse Transcription Kit (Applied Biosystems). Real-time qPCR was performed on an AriaMx Real-Time PCR System (Agilent Technologies), using the DNA binding dye SYBR green (Brilliant III SYBER-GREEN Master Mix). Amplification was performed using the following primers: PARP-1 (5'->3'): F-AACTTGAGCAGATGCCCTCC, R-CCTCTTCGTCCTGGCCATAG; SIRT1 (5'->3'): F-AGAACCAAAGCGGAAA, R-TCCCACAGGAGACAGAAACC; actin (5'->3'): F-TCTGGCACCACACCTTCTA; R-AGGCATACAGGGACAGCAC.; IL6(5'->3') F-TCCTCTCTGCAAGAATTCC, R-

TTGTGAAGTAGGGAAGGCCG; IL1B(5'->3') F-TGCCACCTTTTGACAGTGATG, R-ATACTGCCTGCCTGAAGCTC; TNFα(5'->3') F-TTCTCTTCAAGGGACAAGGCT, R-CTGGAAGACTCCTCCCAGGTAT. Levels of mRNA were normalized concerning levels of actin mRNA. Quantification was performed with the relative $2^{-\Delta\Delta Ct}$ method (34) and values were normalized by β-actin mRNA levels. Dissociation curves are going to be analyzed to verify the purity of products. All samples were run in triplicate.

Western blot.

Hippocampal extracts were resolved by 10% SDS-PAGE and then transferred to PVDF membranes. Blots were blocked for 1 hour at room temperature in Tris-buffered saline (TBS) containing 0.2% Tween-20 and 5% fat-free milk. Overnight incubation with primary antibodies was performed at 4°C using the following concentrations and sources: PARP-1, 1:1000 (120 kDa, Thermo Fisher, cat. No. 39559); Sirt1, 1:1000 (110 kDa, Abcam, cat. No. ab110304); CD38, 1:1000 (42 kDa, Abcam, cat. No. ab216343); BDNF, 1:4000 (28 kDa, Abcam, cat. No. ab108319); GSH groups, 1:1000 (Abcam, cat. No. ab19534); Ryanodine receptor, 1:800 (>250 kDa, Invitrogen, cat. No. MA3-916). Following primary antibody incubation, membranes were incubated for 1.5 hours with HRP-conjugated secondary antibodies and developed using enhanced chemiluminescence (Amersham Biosciences, Bath, UK). To correct for loading differences, membranes were stripped and re-probed with β-actin antibody, 1:10,000 (Abcam, cat. No. ab8227). The films were scanned, and densitometric analysis of the bands was performed using the ImageJ free viewer software. Detection of RyR S-glutathionylation was performed according to Kemmerling et al., (2007). Briefly, the hippocampal tissue was placed in a glass/Teflon homogenizer and 200 µl of lysis solution (in mM: 20 MOPS-Tris, pH 7.0, 300 sucrose, 2 EDTA; 1% NP-40 and 0.1% SDS) were added, plus 1 mM BAPTA and protease inhibitors. Hippocampal extracts were

dissolved (1:1) in 2-fold concentrated non-reducing loading buffer containing urea plus N-ethylmaleimide (12 M urea, 2% SDS, 0.04% Bromophenol blue, 96.4 mM NaH_2PO_4, 34 mM Na_2HPO_4, 10 mM N-ethylmaleimide, plus protease and phosphatases inhibitors), incubated at 60 °C for 20 min and separated by PAGE in 10 % polyacrylamide gels under non-reducing conditions using the Tris-Acetate buffer system. After proteins were transferred for 3 h at 100 V to PVDF membranes (Millipore Corp., Bedford, MA); membranes were blocked at 4 °C overnight in Tris-buffered saline (TBS) (20 mM Tris–HCl, pH 7.6, 140 mM NaCl) containing 5% BSA plus 0.2% Tween-20, and were then incubated at room temperature 3 h with anti-glutathione (anti-GSH) antibody (diluted 1:800 in TBS, 5% BSA, 0.2%Tween-20). After washing membranes were incubated at room temperature with the secondary antibody for 1 h. After washing, membranes were incubated with the secondary antibody for 1 h. Blots were quantified by densitometric analysis using the Quantity One software (Bio-Rad Laboratories, Hercules, CA). Results are expressed as the ratio of anti-GSH/RyR band densities (35).

Statistical analyses

Statistics were performed using Prism 5 (GraphPad Software, Inc.). Values represent Mean ± SEM. The Shapiro-Wilk normality test was applied. For multiple groups, a two-way ANOVA followed by Holm-Sidak's post hoc test, or a Kruskal-Wallis multiple comparison test was used; $p < 0.05$ was considered statistically significant. Further details are provided in the figure legends.
